# The rehabilitation tailor: applying personalized medicine to cancer recovery

**DOI:** 10.3389/fgwh.2024.1254562

**Published:** 2024-09-10

**Authors:** Giulia Bongiorno, Helena Biancuzzi, Francesca Dal Mas, Luca Miceli

**Affiliations:** ^1^ Friuli Riabilitazione Rehabilitation Center, Roveredo in Piano, Italy; ^2^ Department of Economics, Ca’ Foscari University of Venice, Venice, Italy; ^3^ Department of Management, Ca’ Foscari University of Venice, Venice, Italy; ^4^ Collegium Medicum, University of Social Sciences, Łodz, Poland; ^5^ Department of Pain Medicine, IRCCS C.R.O. National Cancer Institute of Aviano, Aviano, Italy

**Keywords:** breast cancer, rehabilitation, electromyography, pain, sport medicine

## 1 Introduction

This journal recently published the launch of a personalized rehabilitation project called “the rehabilitation tailor” (1), at the National Cancer Institute of Aviano, Italy. The program is dedicated to women who have had breast cancer operations. The challenge was to exploit the most modern technologies and create a rehabilitation path that could be adapted to the patient's needs and be carried out remotely, allowing her to be educated and supervised during her oncological recovery.

Specifically, this innovative project provides the possibility of working on several rehabilitation aspects, i.e., pain control, physical exercise for strength and resistance (the Oncology in Motion project) (2–4), and kinematics for reconstructing the movement impaired by surgery.

In the continuation of the path, the Institute's choice was to use co-production as a tool for involving the various stakeholders, to further improve and customize the shoulder analysis algorithm available at the Institute and the rehabilitation path “*in toto*” of these patients. Thanks are also due to the professional athletes who engaged and provided help.

## 2 Pathway description

The project was developed with three macro-phases: the collaboration with a major institution in the sector and scientific research, the generation of two different protocols and related scientific publications, and the pilot application of the protocol to practical cases.

In the first phase, a collaboration was built between the Centro di Riferimento Oncologico (CRO) national cancer institute and the Italian National Olympic Committee (CONI), headquartered in Friuli-Venezia Giulia, Italy, through a scientific convention developed *ad hoc* (5), with the aim of acquiring knowledge on athletes that can then be transferred to patients, mostly regarding the kinematics of the shoulder for pathologies inherent to the shoulder joint itself and for implications for the shoulder due to oncological breast pathology. The choice of this institution was linked to its strength in involving all high-level sports disciplines and in its important measurement technologies not available at the CRO institute. This step allowed the medical personnel involved in the project (mainly a pain doctor and a physiotherapist) to increase their kinematic and electromyographic knowledge. After acquiring the necessary background and tools, we proceeded to measure the movements of athletes involved in speed skating, a cyclical and repetitive sport that potentially has less variability in the execution of the movement than patients burdened by possible interference due to pain and functional limitations, based on the type of treatment received (surgery, radiotherapy, or chemotherapy) (6). Athletes, on the other hand, have an almost constant cyclicity in their movement; therefore, their contribution was invaluable in acquiring the know-how necessary to understand and interpret the more marked kinematic variations typical of patients, starting from a constant basis.

The second phase started with the drafting of the results obtained in Phase 1 and their scientific publication as a prototype of kinematic analysis for professional skaters (7). This analysis protocol, built on the cyclical movement of the skaters, can, through minimal calibrations, be used in almost any cyclical and repetitive sport (e.g., running and cycling) and in the analysis of upper limb movement by modifying the positions of the probes on the muscles investigated, as it is applicable to any type of cyclical and repetitive movement. The choice of this sport has also led to the study of asymmetries in muscle fatigue, as it is one of the very few professional disciplines with asymmetrical movement, leading to a differentiated impact on the muscles of the right and left legs. In addition, in this case, a specific and flexible electromyographic analysis protocol was created and published, with potentially useful repercussions on the asymmetries of women who had undergone breast surgery and afterward found themselves not only having differences in strength but also in resistance to fatigue in the two upper limbs (8).

**Figure 1:**
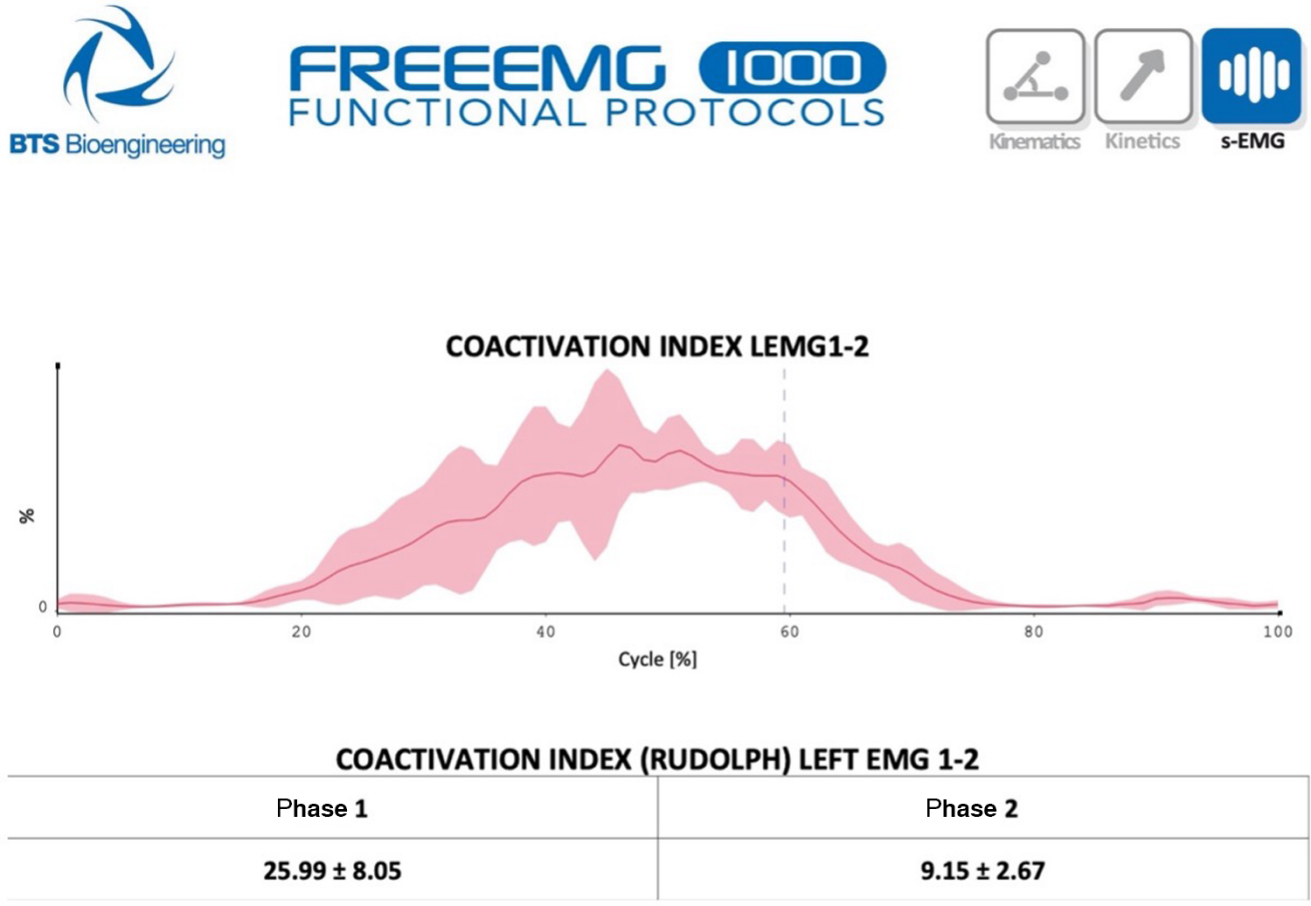
Static and dynamic coactivation indices.

Once this second electromyographic analysis algorithm was also defined, which can be used in the sports and rehabilitation fields like the previous one, we moved on to the third phase, focusing on the theme of muscle coactivations (7). We started from the static formulas described in the literature (9), integrating new dynamic methods into the analysis protocol to understand the coactivation of agonist/antagonist muscles in each phase of the movement (10). As the dynamic formula was developed in the context of the National Institute for Accident Insurance at Work (INAIL), it was decided to also involve this institute in the co-production process through a second scientific agreement stipulated *ad hoc* with the CRO institute (11) (Figure 1). The involvement of INAIL was particularly important in directing the attention of breast operation patients to the post-treatment phase, given that breast cancer disease often affects young women, who need to be reintegrated into work after the etiological treatments (1). To expand the use of movement analysis technologies in rehabilitation, even at home, using the position sensors already present in smartphones, a computer scientist expert in artificial intelligence applications has been involved in the creation of software capable of evaluating the asymmetries and fluidity in movement with objective indices. This software has also been developed for roller speed skating but it can potentially be used for any repetitive movement, such as the rehabilitation movement of the shoulder in women who have undergone breast surgery (4).

## 3 Discussion

The path described presents interesting and innovative aspects in the field of co-production in healthcare, starting from experiences already studied by the authors (4, 12, 13). There were many stakeholders involved, with each contributing to the already existing “Oncology in Motion” project (2). CONI and the athletes analyzed provided the patients with a message of putting in the effort to achieve a goal, with the only difference being that the athletes choose the race they participate in, unlike the patients. The physiotherapist-athlete has combined the fields of sport, research, and health rehabilitation in the same person, spreading these concepts to patients on a daily basis. The engineers and the computer scientist have built a personalized kinematic analysis algorithm based on the most modern knowledge in this area, developing protocols that can be used in other cyclic sports but, above all, in rehabilitation, specifically for the shoulders of women who have undergone breast surgery. The pain doctor has worked to make it possible for women affected by severe pain after surgery to access physiotherapy using the pulsed radiofrequency techniques at his disposal, and he lent himself as a subject for one of the kinematic studies, wearing for the first time turns the skates and testing the goodness of the algorithm in discerning the kinematics differences between the perfect movement (that of the professional athlete, the physioterapist) and the one that can be perfected (that of neophyte skater, the pain doctor himself) (14). The athletes involved, some very young, understood the importance of helping, when healthy, scientific research for the benefit of those suffering from important pathologies and setting an example from a young age. The two co-production experts in the team have provided dignity and scientific solidity to the “Oncology in Motion” path already started but, more importantly, patients suffering from shoulder pathology (15) and women who have undergone breast surgery now have new and peculiar analysis algorithms for their rehabilitation and are aware that they are not alone in their path. An attempt at translating these protocols from the world of sports to breast cancer pathology was first undertaken in 2023, in which a particularly complex case of a patient who had undergone bilateral breast cancer surgery was successfully managed using the algorithms described above, and a personalized rehabilitation plan was created for her (16). In this case, the algorithms for the activation and coactivation of the shoulder joint muscles, along with the study of the fluidity of upper limb movement, using indices derived from the study of the legs of professional roller skaters, led to the identification and quantification of the patient's deficits and the proposal of an effective physiotherapy pathway with targeted exercises and a pain modulation technique through pulsed radiofrequency of the suprascapular nerve. Adapting the path from the world of sports to oncology required an in-depth knowledge of movement kinematics, as the variability in the cyclicity of a professional athlete is significantly lower than that of patients. Therefore, every variation in these latter subjects during motor tasks had to be evaluated with great care, always considering their clinical condition. In particular, the analysis protocol developed for athletes was adapted for patients with breast cancer by modifying the minimum number of repetitions required for each motor task investigated (at least six for each movement, from which average values are then extrapolated), as the variability among patients is greater than that of athletes, who exhibit more homogeneous movements, allowing for fewer repetitions. The study of muscle coactivations in breast cancer patients also included analysis of the pectoralis major muscle, which is of less interest in athletes. Based on kinematic shoulder analysis, we hypothesized a personalized physiotherapy plan primarily targeting the middle trapezius and pectoralis major muscles. The study of muscle coactivations has also led to a better understanding of the effectiveness of pulsed radiofrequency in increasing the synergy between the pectoralis major and latissimus dorsi muscles, primarily through pain control. A limitation to this approach is that not all healthcare institutions have access to the advanced technologies described above, due to costs and the expertise required to use them. As these technologies are also used in several professional sports, hospitals that are not equipped with them can collaborate with their respective national Olympic committees to gain access to the analysis devices available in sports societies. They can also leverage telemedicine to interpret the acquired data by sending them to more experienced rehabilitators, regardless of their location. A possible solution is therefore the creation of a consultancy service that the CRO of Aviano or other hospitals with a good knowledge of the kinematic analysis can offer to users from other locations, by studying selected subjects on-site and providing a sort of “advanced rehabilitation dossier” to the physiotherapists and doctors of patients who have a challenging rehabilitation path. This approach would further strengthen the capacity for networking between national and international oncology centers.

## References

[1] Bongiorno G Biancuzzi H Dal Mas F Bednarova R Miceli L. The rehabilitation tailor: applying personalized medicine to cancer recovery. Front Glob Womens Health. (2022) 3:1–4. 10.3389/fgwh.2022.914302 PMC9314649 35903486

[2] Bednarova R Biancuzzi H Rizzardo A Dal Mas F Massaro M Cobianchi L Cancer rehabilitation and physical activity: the “oncology in motion” project. J Cancer Educ. (2022) 37(4):1066–8. 10.1007/s13187-020-01920-0 33169335

[3] Miceli L Bednarova R Biancuzzi H Garlatti A. Nascita di un percorso riabilitativo in un irccs oncologico del friuli-venezia giulia: “oncology in motion.” Polit Sanit. (2019) 20(2):89–95. 10.1706/3192.31698

[4] Dal Mas F Biancuzzi H Massaro M Miceli L. Adopting a knowledge translation approach in healthcare co-production. A case study. Manag Decis. (2020) 58(9):1841–62. 10.1108/MD-10-2019-1444

[5] CONI e CRO collaborano per la riabilitazione. Centro di Riferimento Oncologico di Aviano (2022). Available online at: https://www.cro.it/it/news/2022/collaborazione-coni.html (accessed July 7, 2023).

[6] Prieto-Gómez V Navarro-Brazález B Sánchez-Méndez Ó De-la-Villa P Sánchez-Sánchez B Torres-Lacomba M. Electromyographic analysis of shoulder neuromuscular activity in women following breast cancer treatment: a cross-sectional descriptive study. J Clin Med. (2020) 9(6):1804. 10.3390/jcm9061804 32531893 PMC7355794

[7] Bongiorno G Biancuzzi H Dal Mas F Fasano G Miceli L. Roller speed skating kinematics and electromyographic analysis: a methodological approach. Sports. (2022) 10(12):209. 10.3390/sports10120209 36548506 PMC9781641

[8] Bongiorno G Biancuzzi H Dal Mas F Miceli L. Evaluation of muscle energy in isometric maintenance as an index of muscle fatigue in roller speed skating. Front Sport Act Living. (2023) 5:1153946. 10.3389/fspor.2023.1153946 PMC10077145 37033883

[9] Rudolph KS Axe MJ Snyder-Mackler L. Dynamic stability after ACL injury: who can hop? Knee Surg Sport Traumatol Arthrosc. (2000) 8(5):262–9. 10.1007/s001670000130 11061293

[10] Ranavolo A Mari S Conte C Serrao M Silvetti A Iavicoli S A new muscle co-activation index for biomechanical load evaluation in work activities. Ergonomics. (2015) 58(6):966–79. 10.1080/00140139.2014.991764 25555042

[11] Centro di Riferimento Oncologico di Aviano. Collaborazioni scientifiche (2022). Available online at: https://www.cro.sanita.fvg.it/it/ricercatori/collaborazioni.html (accessed July 7, 2023).

[12] Dal Mas F Biancuzzi H Massaro M Barcellini A Cobianchi L Miceli L. Knowledge translation in oncology. A case study. Electron J Knowl Manag. (2020) 18(3):212–23. 10.34190/EJKM.18.03.002

[13] Cobianchi L Dal Mas F Massaro M Bednarova R Biancuzzi H Filisetti C Hand in hand: a multistakeholder approach for co-production of surgical care. Am J Surg. (2022) 223(1):214–5. 10.1016/j.amjsurg.2021.07.053 34376274

[14] Bongiorno G Sisti G Dal Mas F Biancuzzi H Bortolan L Paolatto I Surface electromyographic wheel speed skate protocol and its potential in athletes’ performance analysis and injury prevention. J Sports Med Phys Fitness. (2023) 63(10):1093–9. 10.23736/S0022-4707.23.15045-6 37382412

[15] Bongiorno G Bednarova R Biancuzzi H Dal Mas F Rizzardo A Tomasi A Pulsed radiofrequency as a standalone treatment for adhesive capsulitis. Surgeries. (2023) 4(3):335–41. 10.3390/surgeries4030034

[16] Bongiorno G Tomasi A Vigni G Rizzardo A Biancuzzi H Dal Mas F Case report: movement analysis in oncological rehabilitation: proposal of a kinematic and surface electromyographic protocol in breast oncology. Front Hum Neurosci. (2024) 17:1272027. eCollection 2023. 10.3389/fnhum.2023.1272027 38328676 PMC10848327

